# Symptomatic Gallbladder Duplication With Mucocele and Calculi: An Anatomic Variant Diagnosed by CT

**DOI:** 10.7759/cureus.87782

**Published:** 2025-07-12

**Authors:** Quang Dai La, Aiman Baloch, Muhammad Ayub, Sobia Ahmed, Mahrukh Panezai, Farzana Rahim, Francis Pryor, Pari Gul

**Affiliations:** 1 Medicine, The Innovative STEMagazine, College Station, USA; 2 Biology, Texas A&M University, College Station, USA; 3 Medicine, Mekran Medical College, Turbat, PAK; 4 Radiology, Bolan Medical Complex Hospital, Quetta, PAK; 5 Medicine, Lake Erie College of Osteopathic Medicine, Erie, USA; 6 Radiology, Bolan Medical College, Quetta, PAK

**Keywords:** anatomic variant, cholecystectomy, cholelithiasis, computed tomography (ct), congenital biliary anomalies, gallbladder duplication, mucocele

## Abstract

Gallbladder duplicates are generally asymptomatic and discovered incidentally; meanwhile, complications with gallbladder duplication, such as cholelithiasis and mucocele, can result in acute symptoms in the abdominal region with both complicated diagnostic and/or surgical difficulties. This report describes a 51-year-old male patient who presented to our facility with colicky right upper quadrant abdominal pain. Contrast-enhanced CT demonstrated true gallbladder duplication with two separate cystic ducts, one of which was distended secondary to a mucocele from an obstructing calculus. An elective cholecystectomy was performed, with intraoperative inspection revealing that there were two distinct anatomically separate gallbladders, and the two gallbladders demonstrated chronic cholecystitis with multiple calculi noted in both. The patient returned home on postoperative day five without complications. This case demonstrates perhaps the clinically relevant need to offer gallbladder duplication diagnosis, especially in an obstructive pathology context. To make the diagnosis and plan for the surgical procedure, preoperative imaging is required to direct an informed and ultimately safe approach to surgery that includes all intraoperative challenges of this duplicate anatomy and the best treatment to cure the patient.

## Introduction

Gallbladder duplication is an uncommon congenital anomaly with an estimated prevalence of one in 4,000 live births [1]. The duplication of gallbladders presents challenges to diagnosis; most patients have gallbladder duplication discovered incidentally either during imaging or during surgical procedures [2]. Common presentations of gallbladder duplication include typical biliary symptoms like right upper quadrant (RUQ) abdominal pain, typically because of some type of complication like cholelithiasis or mucocele development involving one or both gallbladders [2]. It is important to be aware of the preoperative diagnosis in patients with gallbladder duplication to decrease the risk of surgical complications, as, independently, each duplicate gallbladder may possess its own cystic duct or vascular supply [3]. Harlaftis' classification considers Type I duplication as a split primordial gallbladder with a common cystic duct and Type II as two separate gallbladders with separate cystic ducts. In terms of surgery, when patients with gallbladder duplication assume the risks of recurrence of symptoms and other complications, elective cholecystectomy of both gallbladders is an option [2]. Therefore, preoperative diagnosis is difficult, as imaging suggests duplicated cystic ducts and routinely does not differentiate true duplication from common mimics like choledochal cysts or folds.

This case report describes a case of gallbladder duplication complicated by mucocele development and cholelithiasis in a 51-year-old male patient who presented with colicky left upper quadrant abdominal pain that was diagnosed preoperatively and was successfully treated with elective cholecystectomy.

## Case presentation

A 51-year-old man presented to the surgical emergency department with intermittent colicky RUQ abdominal pain that had persisted for 48 hours. He had not had fever, vomiting, or jaundice. He had no previous episodes of this pain, and his past medical and surgical history was unremarkable.

On examination, the patient had right hypochondrial localized tenderness, with no evidence of peritonitis. Vital signs were stable. Blood tests, including liver function tests and a complete blood count, were normal.

**Figure 1:**
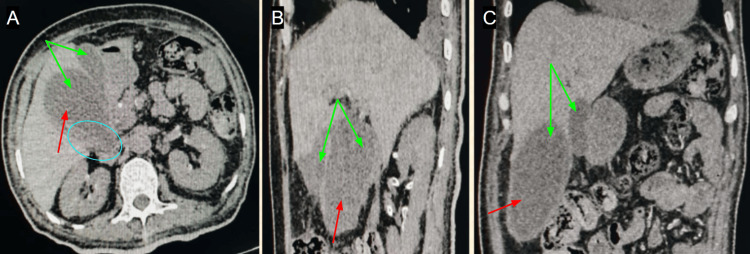
CT (A) axial, (B) sagittal, and (C) coronal reformatted images showing duplicated gallbladder with one of these appearing markedly distended (mucocele), showing dependent calculi. Green arrows: duplicated gallbladder; red arrows: distended gallbladder; and cyan circle: calculi

**Figure 2:**
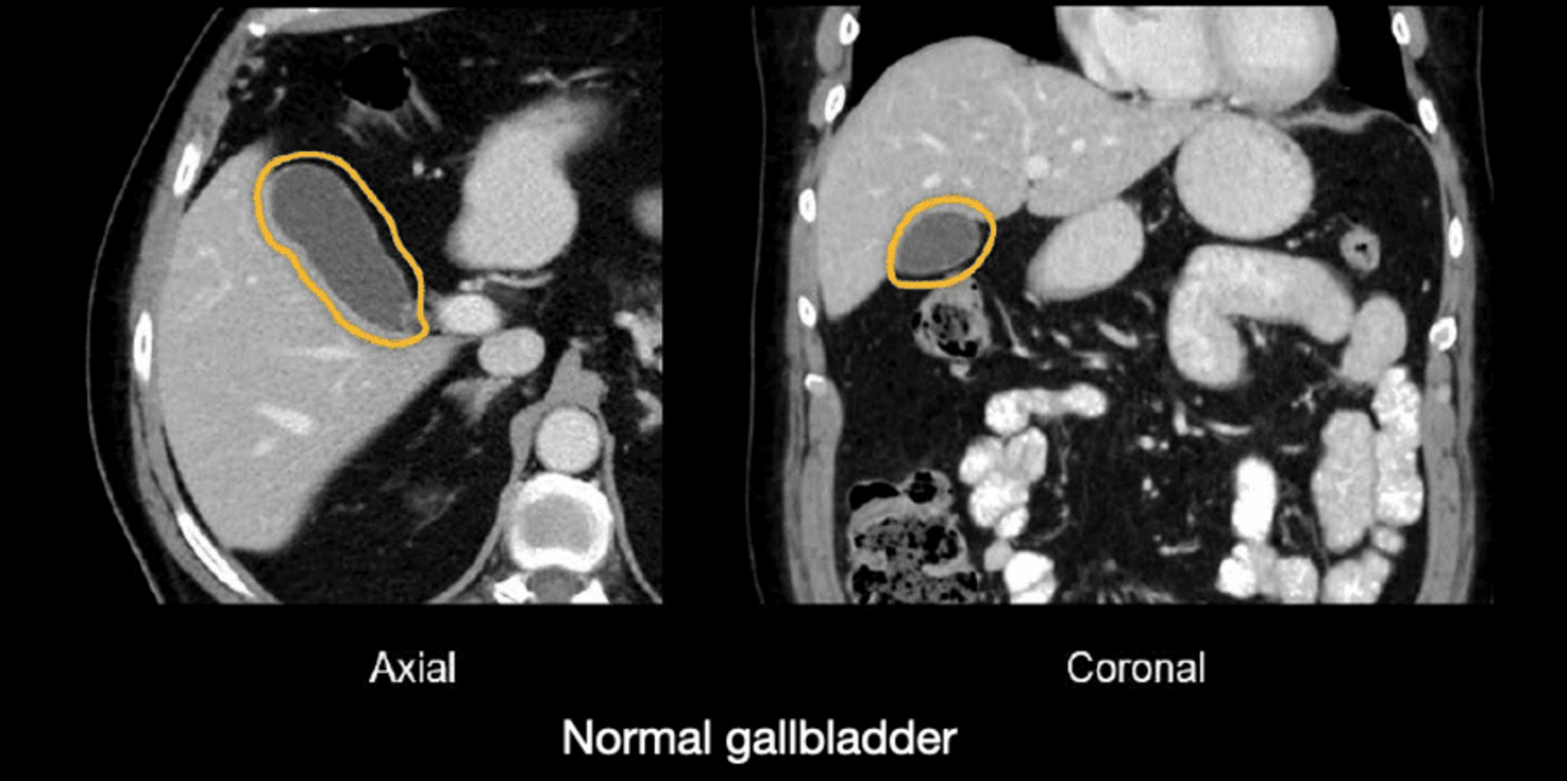
CT axial and coronal images showing normal gallbladder visualization. The image is reproduced from Hartung and Brown (2024) [4] under the CC BY 4.0 license.

Contrast-enhanced CT of the abdomen demonstrated a duplicated gallbladder, one of which was markedly overdistended and had multiple small dependent hyperdense foci consistent with calculi (Figure 1). The duplicated anatomy that was subsequently examined was demonstrated to be formed by two, fully separate gallbladders that had separate cystic ducts. For comparison, Figure 2 depicting a normal gallbladder CT has been included [4].

Elective cholecystectomy was performed. Intraoperatively, we documented two gallbladders with separate cystic ducts, revealing true gallbladder duplication. One gallbladder was distended and enlarged with markedly greater dimensions than the second, attributable to a mucocele secondary to an obstructing stone in its cystic duct. Both gallbladders contained multiple small dependent stones. No intraoperative or postoperative complications occurred.

The patient had an uncomplicated recovery and was discharged on postoperative day five with relevant follow-up instructions. Histopathology confirmed chronic cholecystitis of both gallbladders.

## Discussion

Gallbladder duplication is a rare congenital anomaly first described by Boyden in 1926, with an incidence reported to range from one in 4000 to one in 5000 inhabitants [5]. Gallbladder duplication is a result of an embryologic developmental error occurring in the fifth and sixth weeks of gestation, producing two gallbladders, either having a common cystic duct or two separate cystic ducts, as was the case in our patient [6]. Harlaftis et al. described gallbladder duplication in a classification scheme including two types: Type I are gallbladders with a common cystic duct, and Type II are gallbladder duplications with two separate cystic ducts, matching the intraoperative appearance in our case [7,8].

**Figure 3:**
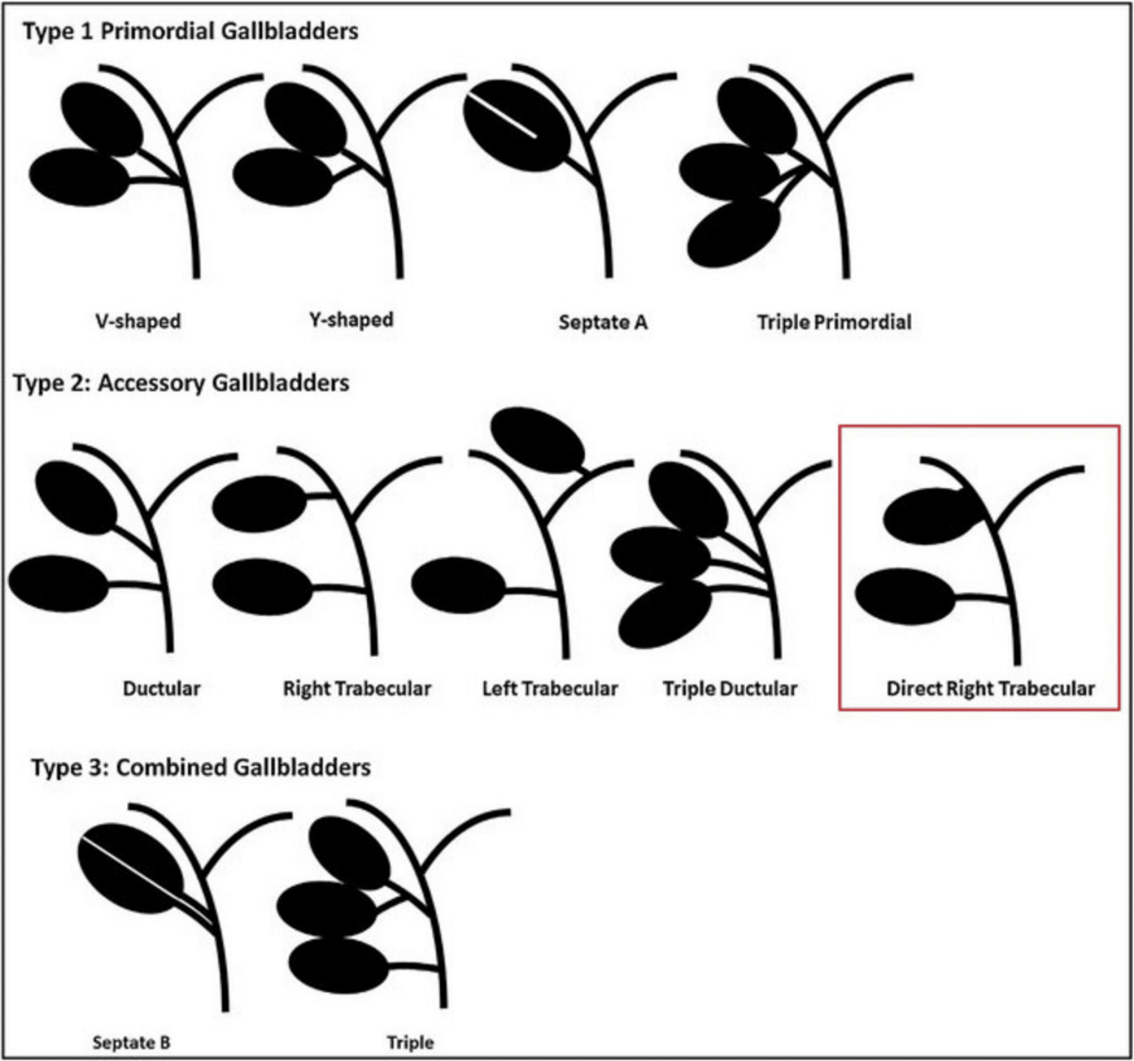
Classification of duplicated gallbladders, including the new variant, showing an intrahepatic gallbladder communicating directly with the right hepatic duct. The image is reproduced from Wong et al. (2018) [11] under the CC BY 4.0 license.

Clinically, with duplicated gallbladders, patients experience symptoms similar to those with normal gallbladders and eventually develop complications such as cholelithiasis, cholecystitis, or mucocele formation that lead to symptoms [9]. Our patient presented with typical RUQ colicky pain and no jaundice or infection, consistent with previously reported cases where gallbladder duplication was complicated by symptoms of cholelithiasis and mucocele [10]. Differential diagnoses for a duplicated gallbladder have been added in Figure 3 to aid clinicians in the diagnosis of a duplicated gallbladder [11].

Preoperative diagnosis is difficult but crucial to avoid surprises in the operating room and prevent bile duct injury. There have been studies suggesting that imaging modalities such as ultrasonography, CT, and magnetic resonance cholangiopancreatography (MRCP) may help with diagnosis, with MRCP providing enhanced visualization of biliary anatomy [12]. In our case, CT with contrast was also useful for discovering the duplicated gallbladder and the obstructing calculi causing mucocele formation. This was in agreement with the literature that supports CT imaging in patients with complex biliary anatomy when MRCP is not available or contraindicated [13].

Surgical management requires discrimination and careful dissection to identify and remove both gallbladders and both cystic ducts to avoid symptoms of recurrence or post-cholecystectomy syndrome [14]. Our patient successfully underwent an elective cholecystectomy with no postoperative complications, which is consistent with reported similar cases [15]. Histopathological investigation revealed chronic cholecystitis in both gallbladders, which has been reported in duplicated gallbladders as a result of bile stasis and recurrent inflammation [16].

## Conclusions

In summary, this case report adds to the relatively sparse but emerging literature regarding duplication of the gallbladder with complications of mucocele and cholelithiasis. It also serves as a reminder of the opportunity to implement thorough preoperative imaging and the awareness of duplicated gallbladder anomalies in surgical planning to prevent unexpected complications.
